# The Dual Role of Macroglia in Glaucoma: Deciphering the Contributions of Astrocytes and Müller Cells to Retinal Neurodegeneration and Neuroprotection

**DOI:** 10.3390/ijms27156895

**Published:** 2026-08-01

**Authors:** Guilherme Ribeiro Teixeira, Ana Gabriela Alves Costa, Ana Carolina de Luca Mattos, Daniel Souza Monteiro de Araújo, Rafael Brito, Karin da Costa Calaza

**Affiliations:** 1 Laboratory of Retinal Neurobiology, Department of Neurobiology, Institute of Biology, Fluminense Federal University, Niterói 242102401, RJ, Brazil; gr_teixeira@id.uff.br (G.R.T.); agalvescosta@id.uff.br (A.G.A.C.); analuca@id.uff.br (A.C.d.L.M.); 2 Graduate Program of Neurosciences, Institute of Biology, Fluminense Federal University, Niterói 242102401, RJ, Brazil; rafaelbrito@id.uff.br; 3 Graduate Program of Biomedical Sciences, Biomedical Institute, Fluminense Federal University, Niterói 242102401, RJ, Brazil; 4 Laboratory of Neuronal Physiology and Pathology, Department of Molecular and Cellular Biology, Institute of Biology, Fluminense Federal University, Niterói 242102401, RJ, Brazil

**Keywords:** intraocular pressure, reactive gliosis, oxidative stress, neuroinflammation

## Abstract

Glaucoma is a leading cause of irreversible vision loss characterized by the progressive degeneration of retinal ganglion cells (RGCs) and structural and biochemical remodeling of the optic nerve head. Although lowering intraocular pressure remains the primary clinical intervention, neurodegeneration often persists, highlighting the complexity and multiple mechanisms involved in the disease’s pathophysiology. In the healthy retina, astrocytes and Müller cells maintain structural integrity, homeostatic balance, and metabolic support. However, sustained pathological stress triggers reactive gliosis, a phenomenon with a dichotomous phenotype. Initially, the macroglial response is adaptive and neuroprotective. Persistent biomechanical and ischemic insults shift this profile into a typically deleterious one, characterized by extracellular matrix remodeling, complement system activation, and heightened neuroinflammation, factors that intensify RGC death. Mechanosensitive pathways, notably Piezo1 and various transient receptor potential (TRP) channels, emerge as critical sensors translating physical stress into these reactive cascades within interconnected multicellular networks. This review examines the crucial role of astrocytes and Müller cells in the dynamic modulation of the retinal microenvironment during glaucomatous progression. Finally, it discusses the therapeutic potential of macroglia-directed pharmacological or gene therapies to reprogram the retinal environment.

## 1. Introduction

Glaucoma comprises a heterogeneous group of progressive optic neuropathies characterized by structural remodeling of the optic nerve head, and progressive loss of retinal ganglion cells (RGCs) and their axons, leading to visual deficits [1,2,3]. It is one of the leading causes of irreversible blindness worldwide, and its prevalence is expected to increase substantially with population aging [3,4]. Although elevated intraocular pressure (IOP) remains the most important modifiable risk factor and the main therapeutic target in clinical practice, glaucomatous neurodegeneration may progress despite apparently adequate pressure control [2,5,6]. This indicates that pressure-dependent and pressure-independent mechanisms converge to drive RGC dysfunction and death.

Over the past decades, glaucoma has increasingly been recognized not only as a pressure-related optic neuropathy but also as a complex neurodegenerative condition involving mechanical stress, vascular dysfunction, impaired axonal transport, mitochondrial dysfunction, oxidative stress, excitotoxicity, immune activation, and neuroinflammation [2,6,7,8]. These mechanisms do not act in isolation. Rather, they occur within a highly specialized retinal and optic nerve microenvironment in which neurons, glial cells, vascular elements, extracellular matrix components, and immune mediators continuously interact [5,8,9].

Glial cells are central regulators of retinal and optic nerve homeostasis. Under physiological conditions, macroglial cells, including astrocytes and Müller glia, provide structural and metabolic support, regulate extracellular ion and neurotransmitter homeostasis, contribute to blood–retinal barrier integrity, and maintain the retinal microenvironment required for neural survival [5,10,11]. Astrocytes are localized in the retinal nerve fiber layer, optic nerve head, and optic nerve, whereas Müller glia span the entire retinal thickness, allowing rapid detection and response to tissue stress [5,9,10,12,13]. In glaucoma, persistent biochemical, metabolic and inflammatory insults profoundly alter macroglial phenotype and function. Although these responses initially promote tissue adaptation and neuronal support, sustained glial activation progressively disrupts retinal homeostasis and contributes to neurodegeneration.

Functional plasticity is one of the defining features of glaucoma. Rather than acting as passive markers of disease progression, astrocytes and Müller cells dynamically shift between homeostatic and reactive phenotypes depending on the magnitude and duration of retinal stress [5,8,13,14]. Understanding the molecular mechanisms governing this transition has become a major focus of glaucoma research, as selective modulation of glial responses may preserve beneficial homeostatic functions while limiting chronic neuroinflammation and neuronal injury [6,13,14]. Therefore, macroglia should not be interpreted simply as passive markers of disease progression, but rather as active participants in the balance between neuroprotection and neurodegeneration.

Interactions between macroglia and microglia further increase the complexity of glaucomatous neuroinflammation. Microglial activation is an early event in several experimental models of glaucoma and may influence astrocytic and Müller glial responses through cytokines, complement components, ATP-dependent signaling, and damage-associated molecular patterns [5,6,8]. Conversely, reactive macroglia can shape microglial recruitment, activation state, and inflammatory output, suggesting that the progression of glaucomatous damage may depend, at least in part, on multicellular glial networks rather than on isolated cellular responses [8,14].

Among the molecular pathways involved in macroglial activation, mechanosensitive ion channels have emerged as important mediators linking elevated IOP to retinal stress responses. Members of the transient receptor potential (TRP) family and PIEZO channels enable macroglia to sense biomechanical and biochemical changes in the retinal microenvironment and have been increasingly implicated in glaucoma pathogenesis [15,16,17].

Understanding the role of macroglia in glaucoma is therefore essential for identifying new therapeutic strategies beyond IOP lowering. While current treatments are mainly directed toward reducing IOP, they do not directly target glial dysfunction, neuroinflammation, or intrinsic RGC vulnerability [2,3,5]. A better characterization of astrocyte and Müller glial responses may reveal mechanisms that can be selectively modulated to preserve beneficial homeostatic functions, while limiting maladaptive inflammatory and neurodegenerative signaling [6,13,14].

In this review, we discuss how macroglial dysfunction contributes to retinal ganglion cell degeneration and optic nerve remodeling in glaucoma. We focus on the distinct and complementary roles of astrocytes and Müller glia in retinal homeostasis, mechanotransduction, oxidative stress, and neuroinflammation, and examine emerging therapeutic strategies, including gene-based approaches, that aim to preserve beneficial glial functions while preventing chronic neurodegeneration.

## 2. Glaucoma

Glaucoma comprises a group of progressive and irreversible optic neuropathies characterized by the degeneration of retinal ganglion cells and their axons, ultimately leading to visual loss. This degenerative process results in structural alterations of the optic nerve, including increased optic disk cupping, a morphological hallmark of the disease [18,19,20]. In glaucoma, optic disk cupping develops progressively as a result of axonal loss and lamina cribrosa remodeling, reflecting the underlying structural damage [21]. The lamina cribrosa exhibits marked glial activation and inflammation, involved with RGC death and optic nerve disintegration [22]. RGCs transmit visual information to brain visual pathways through their axons, which form the optic nerve. Therefore, their degeneration directly compromises visual function. As the leading cause of irreversible visual loss worldwide, glaucoma exerts a significant impact on the quality of life of affected individuals [23].

Glaucoma is recognized as a multifactorial disease, and age is the most relevant unmodified risk factor. Aging is characterized by extensive glial cell alterations, such as reprogramming of glial cells’ gene expression, mitochondrial genomic mutation accumulation, and oxidative stress, that lead to a glial reactivity profile in different areas of CNS [24], similarly observed in glaucomatous retina [25]. Thus, aging enhances retinal susceptibility to increased IOP or ONH damage, probably making the tissue more susceptible to cell death in the glaucoma context [6,25]. Accordingly, a study found a positive association between prior OAG and neovascular age-related macular degeneration [26], another retinal disease associated with aging [27] and glial reactivity [28], suggesting that these diseases could share mechanisms involved in the neurodegeneration. Although sex is not considered a risk factor for developing glaucoma, several studies have described sex-differences in glial cells in CNS physiological conditions, and divergent responses to a variety of pathological insults [29,30]. This is not clearly described in glaucoma, but a growing amount of data suggest that estrogen can protect retinal cells from insult [31,32], potentially via regulation of glial cells [33,34]. Therefore, maybe menopause could act as a risk factor for glaucoma in women, which could explain a higher prevalence of the disease in this group (59%), especially in older women [31]. On the other hand, elevated intraocular pressure is the main modifiable risk factor and the only proven therapeutic target for slowing disease progression [3]. Reduction in IOP is directly associated with decreased glaucoma progression [35,36,37], and topical ocular hypotensive therapy has been shown to be effective in delaying or preventing the development of glaucoma in, at least, part of patients with elevated IOP [36,38]. Indeed, IOP activates several mechanosensitive ions, especially TRPs and PIEZO, in the retina, triggering a number of alterations that generate oxidative stress and inflammation involved in cell death [39]. As it will be discussed later in the review, IOP is also a strong stimulator of glial reactivity.

However, a substantial proportion of patients with glaucoma exhibit disease progression despite adequate IOP control [21,40,41], suggesting the contribution of additional pathophysiological mechanisms [36]. Among these mechanisms, neurodegenerative processes, vascular dysfunction, oxidative stress and inflammatory responses may contribute to retinal ganglion cell death independently of intraocular pressure, reinforcing the complexity of glaucoma pathophysiology and the need for complementary therapeutic approaches [2,42]. Accordingly, increasing evidence points to the involvement of glial-mediated mechanisms in glaucoma pathogenesis, since alterations in retinal glial homeostasis have been associated with retinal ganglion cell degeneration and functional visual deficits even under normotensive conditions [14]. In this context, inflammation and oxidative stress have been implicated as key mediators of RGC degeneration. Ischemic events may trigger an inflammatory response characterized by the production of pro-inflammatory mediators and the activation of resident inflammatory cells, contributing to the establishment of a neurotoxic microenvironment that promotes RGC dysfunction and death [2]. Understanding IOP-dependent and -independent mechanisms may complement existing therapeutic strategies or enable the development of novel approaches, contributing to slowing glaucoma progression and preserving visual function.

### Oxidative Stress and Inflammation in Glaucoma

Oxidative stress is a condition where the production of reactive oxygen/nitrogen species (ROS/RNS) overcomes the antioxidant cell defenses [43]. Even in physiological conditions, ROS is present and acts as a signal to a number of homeostatic mechanisms [43]. ROS can be derived from oxygen molecules in the mitochondrial electron chain or by the NADPH oxidase (NOX) enzyme, producing superoxide radicals (O_2_^∙−^), a highly reactive oxygen species with one unpaired electron. Superoxide radicals can combine with nitric oxide (NO), generating peroxynitrite, the main RNS. A non-radical ROS, hydrogen peroxide (H_2_O_2_), can arise from superoxide radicals by the action of superoxide dismutase (SOD). In an oxidative stress condition, even highly reactive radicals can be produced from H_2_O_2_, hydroxyl radicals (OH^∙^). Cells have a myriad of antioxidant defenses, enzymatic or non-enzymatic, that control the balance of physiological signaling ROS/RNS and oxidative stress state. Among them, we can highlight SOD, catalase, peroxiredoxins and glutathione peroxidases as enzymes that detoxify ROS, and glutathione as the main endogenous non-enzymatic antioxidant of the CNS, working together with melatonin, carotenoids and others [44].

ROS/RNS directly react with proteins, lipids, and nucleic acids [45,46], altering their function and leading to damage through a number of mechanisms [47]. Elevated ROS levels, a common feature of neurodegenerative diseases, have been linked to mitochondrial dysfunction, protein misfolding, and disruptions in cellular redox homeostasis [46,48]. Aging increases susceptibility to the accumulation of oxidative damage in cells and tissues, a process that has been linked to cellular senescence and glaucomatous neurodegeneration [49,50]. Additionally, disruption of cellular redox balance may induce a state of microglial hyperactivation, promoting the release of pro-inflammatory mediators such as TNF-α, as well as activation of inflammatory pathways involving NF-κB, nitric oxide synthase, and cyclooxygenase-2 [49]. In glaucoma, alterations in vascular perfusion and reduced oxygen supply may exacerbate oxidative stress, contributing to retinal ganglion cell loss and disease progression [51].

Alterations in ocular vascular autoregulation have been implicated in glaucoma pathophysiology, favoring episodes of reduced ocular perfusion followed by reperfusion, a process associated with the generation of oxidative stress [52,53]. Ocular vascular dysfunction is further characterized by endothelial dysfunction, impaired neurovascular coupling, increased endothelin-1 (ET-1) signaling, and reduced ocular blood flow, all of which contribute to retinal hypoxia and glaucomatous neurodegeneration [53,54]. Persistent hypoxia activates hypoxia-inducible factor-1α (HIF-1α) signaling and promotes the expression of vascular endothelial growth factor (VEGF), while also enhancing the production of inflammatory mediators, including NO, TNF-α, and interleukins, thereby amplifying oxidative stress, inflammation, and neuronal damage [55,56]. Therefore, vascular dysregulation and reduced oxygen supply may exacerbate oxidative stress, contributing to retinal ganglion cell loss in glaucoma.

The inflammatory response in the CNS exhibits a dual role, initially exerting neuroprotective effects through the removal of cellular debris and maintenance of tissue homeostasis. However, when persistent, this response may become dysfunctional, contributing to the inhibition of reparative processes and progression of neuronal damage [57]. In glaucoma, this transition has been associated with chronic activation of glial cells and the maintenance of a pro-inflammatory environment [58], contributing to neuronal degeneration. There are three types of glial cells in the retina: (a) Müller cells, a macroglial cell and the majority retinal glial cell; (b) astrocytes, a macroglial cell with restricted but strategic localization near ganglion cells; and (c) microglia, myeloid-originated cells which invade the tissue during development.

## 3. Astrocytes in the Healthy and Glaucomatous Retina

### 3.1. Distribution and Physiological Functions of Astrocytes

**Figure 1:**
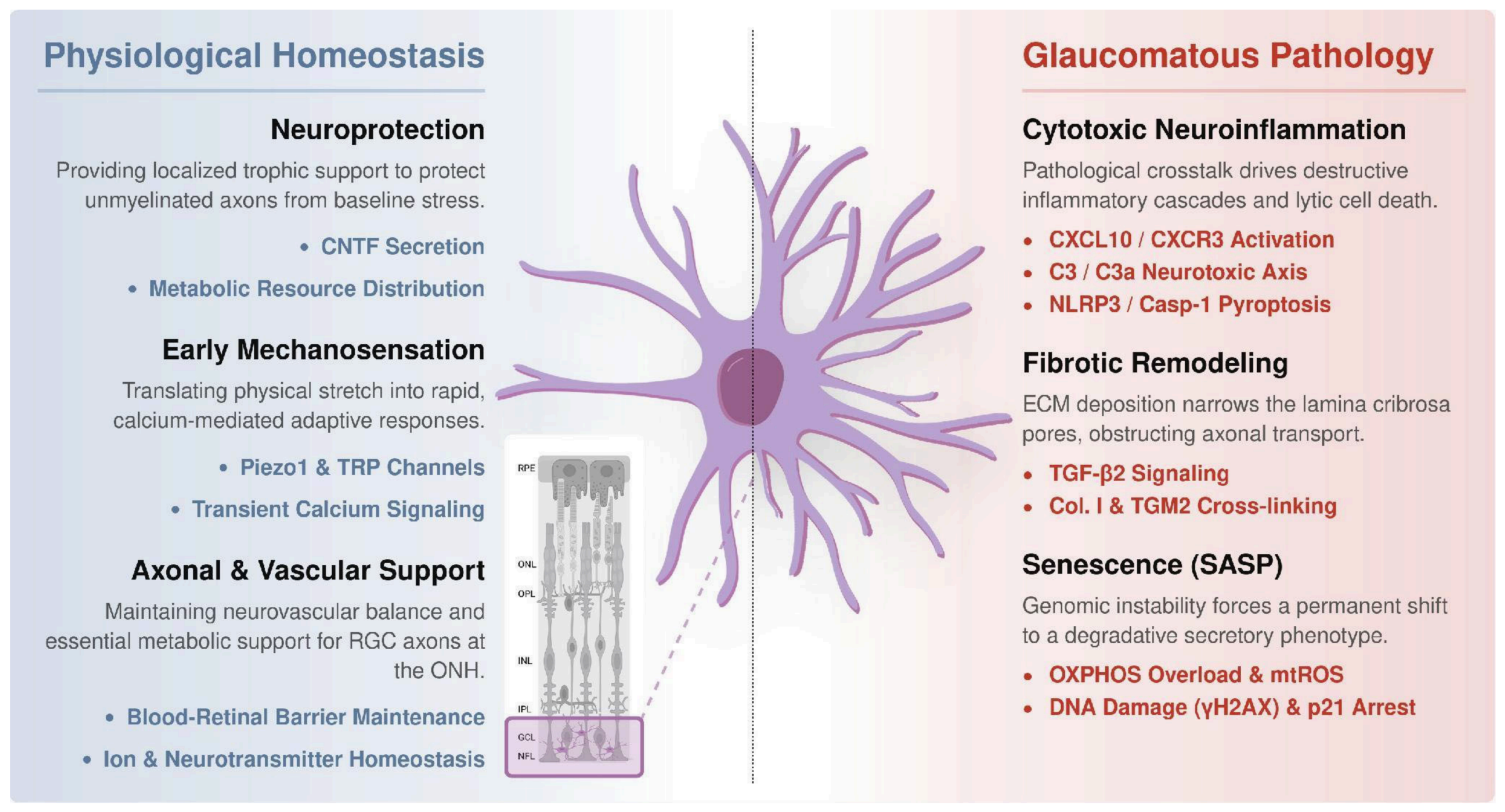
Phenotypic transition of astrocytes in glaucoma. The central conceptual graphic employs a split-cell rendering to directly contrast the two functional states. Under physiological homeostasis (**left side**), astrocytes provide essential neurovascular support, early mechanosensation, and neuroprotection to unmyelinated retinal ganglion cell axons. Sustained biomechanical and oxidative stress (**right side**) drives a pathological collapse characterized by a senescence-associated secretory phenotype (SASP), fibrotic remodeling of the lamina cribrosa, and the activation of cytotoxic neuroinflammatory cascades, including the C3/C3a neurotoxic axis. The central dotted line divides the astrocyte morphology to contrast the two functional states, with the blue background representing physiological homeostasis and the red background indicating glaucomatous pathology. Inset created in BioRender. Calaza, K. D. (2026), https://BioRender.com/5grmlb1 (accessed on 27 July 2026).

Astrocytes form a vital support network for the inner retina and the optic nerve head (ONH) [59], offering continuous mechanical and biological support (Figure 1) [60]. Embryologically, they derive from neuroepithelial cells in the ventricular zone, where radial glial cells act as progenitors that, following neurogenesis, spatially differentiate to produce astrocyte populations [61]. Early in the glial lineage, tripotent glial-restricted progenitors can also give rise directly to type-1 astrocytes, astrocyte-restricted progenitors, and bipotent O2A cells; the latter can further differentiate into type-2 astrocytes [62].

Within the mature retina, the anatomical distribution of astrocytes is highly localized, strictly confined to the innermost strata comprising the ganglion cell layer (GCL) and the retinal nerve fiber layer (RNFL) [63]. Restricted to this microenvironment, they form a dense plexus that interacts directly with retinal ganglion cell axons and the local vasculature. Here, they orchestrate neurovascular balance, resource distribution, and metabolic support (Figure 1) [64]. Classically, these cells are fundamental for maintaining ion and neurotransmitter homeostasis, modulating synapses, and upholding the blood–retinal barrier (Figure 1) [65]. Furthermore, some subpopulations play central roles in coordinating immunological and inflammatory responses against tissue adversities [66], while also secreting crucial trophic factors—such as ciliary neurotrophic factor (CNTF)—that protect RGCs from oxidative damage (Figure 1) [59,67].

### 3.2. Biomechanical Stress and Structural Remodeling

Beyond the retinal boundaries, the optic nerve head acts as a critical transition region. Here, RGC axons converge and pass through a dense astrocytic meshwork in an unmyelinated state, heightening their metabolic vulnerability and making them critically dependent on local astrocytic support before acquiring myelin in the retrolaminar optic nerve [68]. This dense astrocytic meshwork at the ONH represents the primary site of initial RGC injury in glaucoma [68]. The transition of astrocytes to a pathological state is primarily driven by early biomechanical stress (usually from elevated IOP), which exerts tension, compression, and shear forces on the lamina cribrosa [69]. Astrocytes detect these continuous loads via mechanosensitive ion channels, such as Piezo1 and TRPs (particularly the TRPC and TRPV subfamilies), translating physical stretch into a rapid, calcium-mediated reactive response [39,70,71].

**Figure 2:**
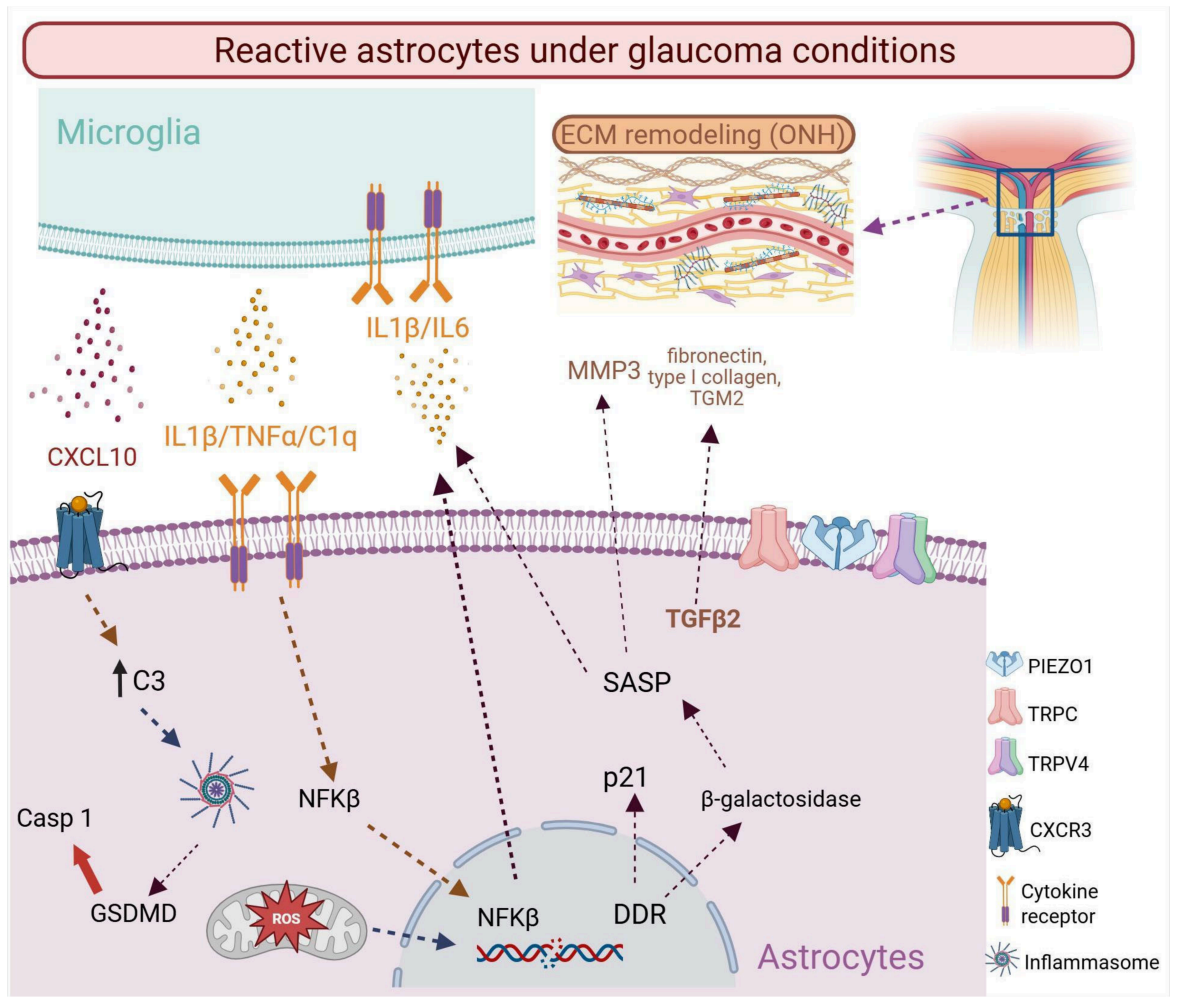
Molecular changes undergone by reactive astrocytes in glaucoma. Microglial cells, in a pro-inflammatory profile, release molecules such as IL-1β, TNF-α, and C1q, via cytokine receptors and NF-ΚB activation, inducing the shift of astrocytes to a neurotoxic phenotype. In this scenario, and also in an NF-κB-dependent manner, astrocytes begin secreting inflammatory mediators into the retinal microenvironment, thereby reinforcing and sustaining the local inflammatory state. The chemokine CXCL10 is also elevated in glaucomatous tissue; by interacting with the CXCR3 receptor, it stimulates astrocytic expression of C3, contributing to the chronicity of the injury and subsequent neuronal loss. The convergence of these pathways culminates in the activation of the NLRP3 inflammasome, which recruits caspase-1. This enzyme cleaves gasdermin D (GSDMD), triggering pyroptosis, resulting in astrocyte lysis and the release of cytoplasmic contents into the nerve fiber layer and optic nerve head (ONH), thereby exacerbating neurodegeneration. Concurrently, the chronic oxidative stress inherent to the pathology causes severe DNA damage, triggering the DNA damage response (DDR) and upregulating the expression of p21 and β-galactosidase. This genotoxic stress drives astrocytes into a state of cellular senescence and the associated secretory phenotype (SASP); the continuous release of cytokines, metalloproteinases and remodeling molecules from this state directly contributes to tissue deterioration of the microenvironment in which these cells are present. In the diagram, dashed arrows indicate downstream signaling pathways, molecular translocations, or cellular secretions, while solid arrows represent direct activation, cleavage, or upregulation. The colored dots represent secreted extracellular mediators. The greenish and purple backgrounds designate the microglial and astrocytic compartments, respectively. Created in BioRender. Calaza, K. D. (2026), https://BioRender.com/2c8ja26 (accessed on 27 July 2026).

Reactive astrogliosis also induces severe extracellular matrix (ECM) remodeling (Figure 2). Mediated by TGF-β2 signaling, reactive astrocytes increase the deposition of macromolecules such as type I collagen and fibronectin, while upregulating tissue transglutaminase (TGM2) to cross-link this newly remodeled extracellular matrix within the lamina cribrosa (Figure 1 and Figure 2) [72]. These stressed astrocytes exhibit process retraction, undergo somatic hypertrophy [73] and, eventually, detach from their basal lamina [74]. This remodeling narrows the radial pores of the lamina cribrosa, obstructing axonal transport and resulting in the progressive optic disk cupping, characteristic of glaucomatous progression [75,76].

### 3.3. Oxidative Stress and Senescence

Alongside this structural remodeling, the transcriptional reprogramming of reactive astrocytes imposes a bioenergetic burden. To meet this ATP demand, ONH astrocytes shift from their basal metabolism to a heavy reliance on oxidative phosphorylation (OXPHOS) [77]. While this metabolic shift maximizes energy production needed for their reactive state, the mitochondrial electron transport chain inevitably leaks electrons, generating a secondary wave of mitochondrial reactive oxygen species (mtROS) as a by-product that exacerbates the ongoing oxidative stress [77]. Initially, there is an upregulation of the antioxidant defense system (such as the glutathione and peroxiredoxin systems) in astrocytes, which could help to buffer oxidative stress, as well as an increase in proteasomal activity that could clear oxidized proteins [77]. However, chronic glaucomatous insult eventually exhausts these defenses, triggering widespread lipid peroxidation and irreversible protein nitration/oxidation. This oxidative collapse destroys the astrocytic cytoskeleton and permanently compromises the metabolic supportive capacity that these cells provide to adjacent RGC axons [77,78].

The consequences of this oxidative stress permanently alter the astrocytes through genomic instability and cellular senescence. In vivo transcriptomic profiling of ONH astrocytes in experimental glaucoma demonstrates that following the initial oxidative burden, astrocytes significantly upregulate pathways associated with the senescence-associated secretory phenotype (SASP) (Figure 1) [77]. The mechanistic basis for this transition has been further mapped utilizing human and murine primary astrocyte cultures exposed to oxidative stressors [79]. These studies confirm that chronic oxidative damage directly induces severe DNA lesions, triggering the DNA damage response (DDR) as evidenced by the accumulation of γH2AX and 53BP1 within the astrocytic nuclei (Figure 2) [79]. This genomic stress forces the reactive astrocytes into a permanent cell cycle arrest, characterized by the upregulation of p21 and β-galactosidase, and locks them into the SASP state (Figure 1 and Figure 2) [79]. These senescent astrocytes become chronic sources of tissue degradation, secreting high levels of matrix metalloproteinases, such as MMP3, and perpetuating the release of inflammatory cytokines like IL-6 and IL-1β (Figure 2) [79]. Through this ROS-driven senescence mechanism, astrocytes abandon their supportive role entirely, actively deteriorating the ONH microenvironment and cementing the progression of glaucomatous neuropathy.

### 3.4. Cytotoxic Shift and Neuroinflammation

Triggered by this oxidative burden and cellular senescence, reactive astrogliosis establishes a self-sustaining neurotoxic shift that perpetuates independently of ongoing IOP elevation, driven by retinal-derived chemokine signaling that serves as a primary molecular trigger for a cytotoxic shift [13]. Following early ocular stress, such as elevated IOP, the injured retinal tissue (specifically RGCs and activated resident microglia) locally upregulates specific chemokines, most notably CXCL10 (Figure 2) [80]. By binding to the CXCR3 receptor on astrocytes, CXCL10 directly drives their polarization toward a neurotoxic profile, primarily by inducing the expression of complement component 3 (C3) (Figure 1) [13,80]. This astrocyte-derived C3 is cleaved into the C3a anaphylatoxin, which specifically binds to the C3aR receptor that becomes concurrently upregulated on adjacent RGCs, establishing a toxic neuroglial crosstalk that synergistically accelerates neuronal apoptosis (Figure 1) [80]. Simultaneously, sustained inflammatory signaling triggers the assembly of the NLRP3 inflammasome within the cytoplasm of reactive astrocytes, activating caspase-1 to cleave Gasdermin D (GSDMD) (Figure 2) and initiate pyroptosis, an inflammatory lytic form of programmed cell death that ruptures the glial membrane and spills extensive cytotoxic contents directly onto the unmyelinated axons [80]. This continuous signaling establishes a localized crosstalk that perpetuates neuropathy in normal-tension glaucoma (NTG) or even after successful IOP reduction. Ischemia–reperfusion, mitochondrial dysfunction, and cytokine-mediated neuroinflammation serve as potent secondary triggers [81,82]. Regardless of the initial insult, the resulting reactive astrogliosis compromises the vital metabolic support provided to the axons, creating a pro-inflammatory and oxidative microenvironment that dictates irreversible glaucomatous neurodegeneration [68,83].

Ultrastructural analyses from an experimental model of ocular hypertension demonstrate how early these neurodegenerative events can occur. With perilimbal-plexus cauterization, transmission electron microscopy showed progressive axonal disorganization, vacuolization of the myelin sheath, and disruption of the nodes of Ranvier [84]. The mechanical stress triggers a robust reactive glial response within the myelinated portion of the optic nerve, characterized by an upregulation of the astrocytic marker GFAP at 48 h and a subsequent accumulation of Iba1-positive microglia/macrophages by 72 h, demonstrating that macroglial and immune remodeling extend rapidly beyond the optic nerve head to propagate the neurotoxic shift [84].

The neuroinflammatory cascade triggers a profound phenotypic shift in astrocytes across a complex and multidimensional spectrum. They exhibit immense heterogeneity, continuously integrating microenvironmental cues to adopt varied transient states before ultimately committing to a pathogenic profile [85]. This polarization towards a cytotoxic state is largely orchestrated by an immune crosstalk with resident microglia, polarized to a pro-inflammatory profile [86]. These activated microglia secrete mediators (IL-1α, TNF-α, and C1q), which seem to be both necessary and sufficient to drive astrocytes into a cytotoxic state (Figure 2) [87]. At the molecular level, this reactivity is regulated by the transcription factor NF-κB, a master regulator of inflammatory and immune responses. Upon stimulation by the microglial mediators, astrocytic NF-κB translocates to the nucleus and drives the gene regulation of cytotoxic mediators, adhesion molecules and immune receptors [88,89].

Persistent inflammatory signaling progressively shifts astrocytes toward a neurotoxic phenotype characterized by the secretion of inflammatory mediators, complement activation, and loss of metabolic support. In this chronic state, they begin to actively secrete cytotoxic factors and cytokines, such as IL-1β and IL-6, often through NLRP3 inflammasome activation, deeply dependent on NF-κB signaling [90,91]. This signaling causes RGCs, already weakened by mechanical strain, to become hypersensitive to the astrocyte-derived harmful signals, leading to accelerated apoptosis [87]. Furthermore, the aberrant upregulation of MHC-II on reactive astrocytes suggests they act as unconventional antigen-presenting cells, potentially driving a localized autoimmune response that sustains neurodegeneration even after IOP stabilization [92], inducing a cell death cycle driven by chemokines and the complement system.

These experimental observations are corroborated by human clinical data. Post-mortem analyses of glaucomatous human retinas and optic nerves confirm widespread glial reactivity, characterized by a significant upregulation of GFAP, p-ERK, and TLR3 [93]. Furthermore, integrated transcriptomic studies identify astrocyte-associated inflammatory genes, such as *SERPINA3* and Lipocalin-2 (*LCN2*), as central pathogenic hubs in primary open-angle glaucoma (POAG) patients [94]. *LCN2* upregulation is closely tied to amyloid-beta (Aβ) toxicity, highlighting a complex neurodegenerative network. This localized tissue damage translates into measurable fluid biomarkers: multiplex assays demonstrate that GFAP, alongside Aβ and the axonal damage marker neurofilament heavy chain (NfH), are significantly elevated in the aqueous humor of POAG patients [95]. In normal-tension glaucoma, while markers of amyloid and axonal pathology (Aβ and NfH) are significantly increased, GFAP levels exhibit a strong upward trend (*p* = 0.058) rather than strict statistical significance [95]. Within the broader glaucomatous spectrum, these elevated intraocular GFAP concentrations negatively correlate with the visual field index and retinal nerve fiber layer thickness, establishing GFAP not merely as a marker of astrocytic damage, but as a clinical indicator of disease severity [95].

Collectively, these findings demonstrate how persistent biomechanical stress progressively converts astrocytes from homeostatic regulators into amplifiers of chronic neuroinflammation. This reactivity is not isolated; astrocytes are physically coupled to Müller cells via Connexin-43 gap junctions [92]. While essential for homeostasis, this connection becomes a pathological conduit that propagates toxic signals, scaling focal optic nerve head injury into a widespread retinal pathology [5]. Therefore, therapeutically decoupling this neurotoxic network, by targeting the SASP, inhibiting specific gap junctions, or modulating the microglial–astrocytic crosstalk, can represent a highly promising path for neuroprotection in the glaucomatous context.

## 4. Müller Glia in the Healthy and Glaucomatous Retina

### 4.1. Müller Cells in Physiological Conditions

**Figure 3:**
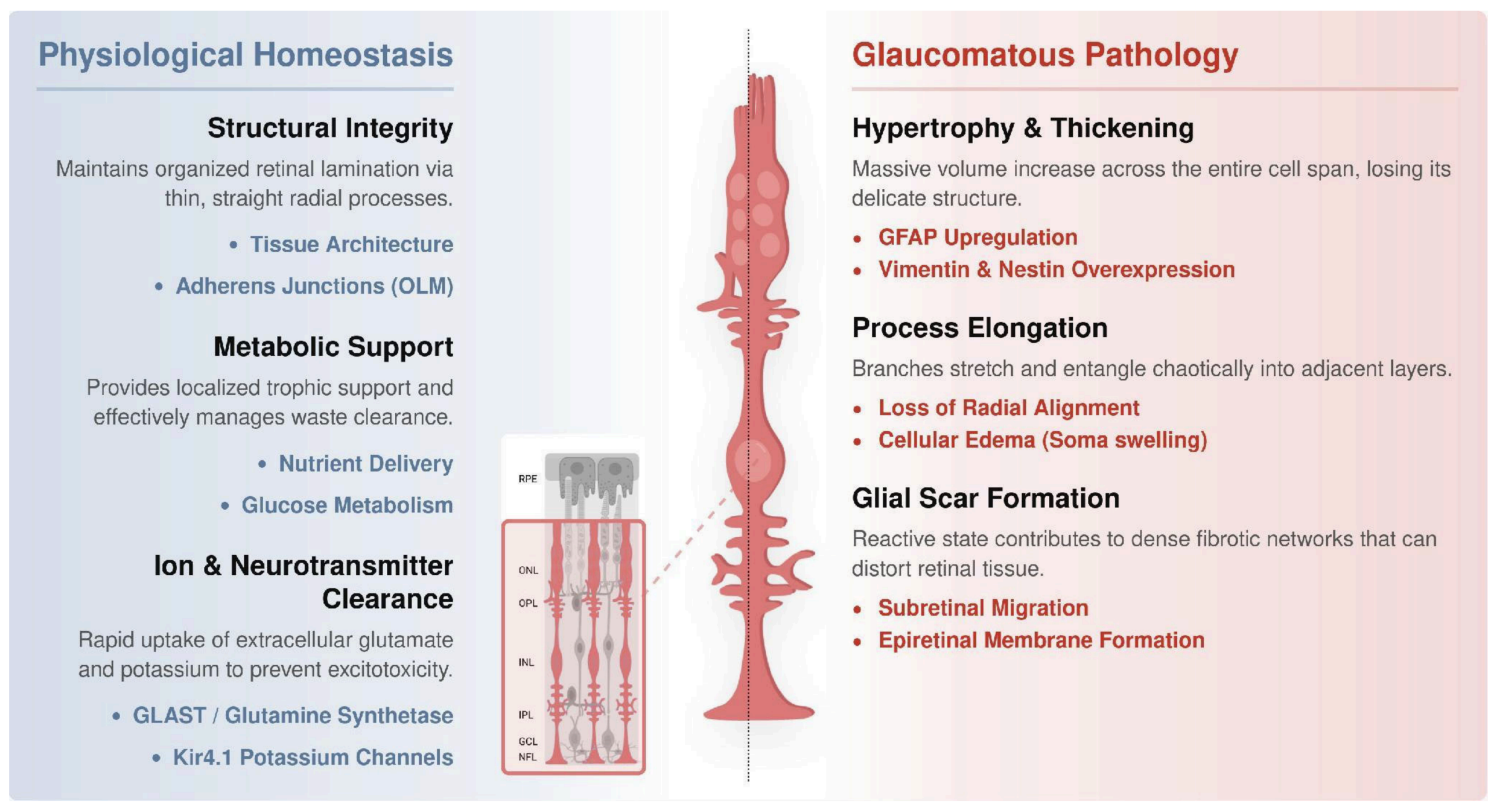
Pathological changes of Müller glia under stress. Using a mirrored split-cell illustration, the figure contrasts the healthy and reactive profiles of a single cell. In a healthy retina (**left side**), Müller cells maintain structural architecture (forming the outer limiting membrane, OLM), offer metabolic support, and prevent excitotoxicity through rapid glutamate and potassium clearance (via GLAST, GS, and Kir4.1 channels). Glaucomatous stress (**right side**) triggers a reactive gliosis marked by profound cellular hypertrophy, GFAP overexpression, elongation of radial processes with soma swelling, and the formation of fibrotic glial scars that disrupt retinal integrity. The central dotted line divides the Müller cell morphology to contrast the two functional states, with the blue background representing physiological homeostasis and the red background indicating reactive gliosis under glaucomatous stress. Inset created in BioRender. Calaza, K. D. (2026), https://BioRender.com/b42xil1 (accessed on 27 July 2026).

Müller glia constitute the predominant macroglial population in the retina, accounting for approximately 90% of retinal glial cells, and are essential for maintaining retinal architecture and function under physiological conditions (Figure 3) [96,97]. They exhibit a unique radial morphology that spans the entire retinal thickness. Due to this peculiar organization, Müller cells interact with all retinal cell types, enabling them to usually be the first glial cell type to detect retinal damage [98]. The Müller radial cell processes in the outer retina form critical junctional complexes with the inner segments of photoreceptors to establish the outer limiting membrane (OLM) [99]. This structure acts as an important macromolecular barrier (Figure 3) and is intrinsically linked to fluid homeostasis, where Müller cells actively regulate subretinal fluid absorption via aquaporin-4 (AQP4) water channels [99].

Beyond structural support, these cells play a central role in retinal metabolic compartmentation, acting as the principal retinal source of glutamine synthetase (GS) and rapidly clearing excitatory glutamate from the synaptic cleft, thus preventing excitotoxicity, and converting it into non-toxic glutamine, which is exported back to neurons, allowing continuous neurotransmission (Figure 3) [100]. Additionally, they provide metabolic support by supplying lactate to retinal neurons, fueling ATP production via the tricarboxylic acid (TCA) cycle, meeting the retina’s fluctuating and high energy demands and participating in the visual cycle through pigment regeneration [99,100], forming an integrated support network that contributes to RGC survival (Figure 3). To sustain the intense bioelectric activity of the retina, these cells ensure tight control over ionic and fluid homeostasis. During continuous neuronal firing, they actively buffer extracellular potassium levels, a process dependent predominantly on the inwardly rectifying potassium channel Kir4.1 (Figure 3) [101]. The functional coupling of Kir4.1 and AQP4 is critical for coordinating ion and fluid clearance; experimental ablation of this delicate balance (the downregulation of Kir4.1 accompanied by AQP4 upregulation) is a major contributor to cytotoxic Müller cell edema [102].

Complementing this physiological regulation, Müller glia play a central role in the retinal antioxidant response. By utilizing systems such as the cystine/glutamate antiporter (xCT) to import necessary precursors, they sustain the intracellular synthesis of glutathione (GSH), providing a major component of retinal antioxidant defense against ROS continuously generated by the high mitochondrial activity of the outer retina [103,104]. Alongside this redox regulation, Müller cells provide an important neuroprotective mechanism by secreting neurotrophins (Figure 3), most notably brain-derived neurotrophic factor (BDNF). By binding to the TrkB receptor on RGCs, Müller-derived BDNF activates the PI3K/AKT signaling cascade, promoting neuronal survival and mitigating axonal and dendritic degeneration under toxic stress [105]. The disruption of these homeostatic mechanisms, marked by Kir4.1 depletion, glutathione depletion, and neurotrophin signaling failure, contributes to the transition from physiological support to reactive gliosis.

### 4.2. Early Reactive Gliosis and Mechanotransduction

Although Müller glia are exposed to the same biomechanical environment described for astrocytes, their response is distinguished by profound alterations in retinal homeostasis rather than by structural remodeling of the optic nerve head. Because these cells extend across the entire retinal thickness, their activation has widespread consequences for neurotransmitter recycling, potassium and water homeostasis, antioxidant defenses, and neurotrophic support [10,106]. Initially, Müller cell activation represents an adaptive and neuroprotective response. One of the earliest hallmarks of this process is the upregulation of GFAP (Figure 3), which extends from its basal expression in astrocytes to the radial processes of Müller glia [107]. During this compensatory stage, Müller cells enhance their homeostatic functions by increasing neurotrophic factor secretion, promoting glutamate uptake and recycling, strengthening antioxidant defenses, and improving potassium buffering capacity [10,106,108]. Consistent with this protective phenotype, increased expression of pigment epithelium-derived factor (PEDF), BDNF, and endogenous antioxidants such as glutathione and ascorbate contributes to limiting early glaucomatous damage [106,109,110]. Together, these adaptive responses temporarily preserve retinal homeostasis before prolonged glaucomatous stress drives Müller cells toward a dysfunctional and pro-inflammatory phenotype.

Beyond these early homeostatic adaptations, several intracellular signaling pathways determine whether Müller cells maintain their protective phenotype or progress toward chronic gliosis. Among these, the Hippo pathway effector Yes-associated protein (YAP) has emerged as an important regulator of Müller cell homeostasis [111]. In experimental glaucoma, despite its classic pro-fibrotic and proliferative role in other tissues [112,113], early YAP activation appears to exert predominantly neuroprotective effects by promoting the transcription of survival-related genes, including the anti-apoptotic protein Bcl-xL, thereby preserving mitochondrial integrity and supporting adjacent retinal ganglion cells following retinal injury [111]. Consistent with this protective role, conditional deletion of YAP specifically in Müller glia results in impaired water and glutamate clearance, leading to severe retinal dysfunction and spontaneous glaucoma-like alterations [114]. Under chronic mechanical stress, such as sustained elevated IOP, YAP’s mechanosensory activity becomes dysregulated. At this point, YAP shifts from a homeostatic survival factor to a driver of pathological fibrosis by upregulating extracellular matrix components, contributing to late-stage glaucomatous remodeling and maladaptive gliosis [114]. These findings suggest that early YAP activation represents an adaptive mechanism that supports Müller cell function, whereas persistent glaucomatous stress may eventually overwhelm or dysregulate this protective pathway.

As homeostatic mechanisms become progressively compromised, persistent activation of signaling pathways downstream of mechanosensitive channels—including TRPV4 and PIEZO1—reinforces Müller cell reactivity and promotes the transition toward a pro-inflammatory phenotype [115,116,117]. Rather than initiating mechanotransduction itself, sustained activation of these pathways amplifies intracellular inflammatory signaling, particularly through IL-6/STAT3 and ERK1/2, resulting in cytoskeletal remodeling, cellular hypertrophy, proliferation, and increased expression of GFAP and nestin (Figure 3) [107,118,119,120]. Progressive gliosis is accompanied by dedifferentiation and loss of essential homeostatic functions, including the downregulation of glutamine synthetase, thereby uncoupling Müller glia from retinal neurons and shifting their role from neuroprotection to neurotoxicity [121].

**Figure 4:**
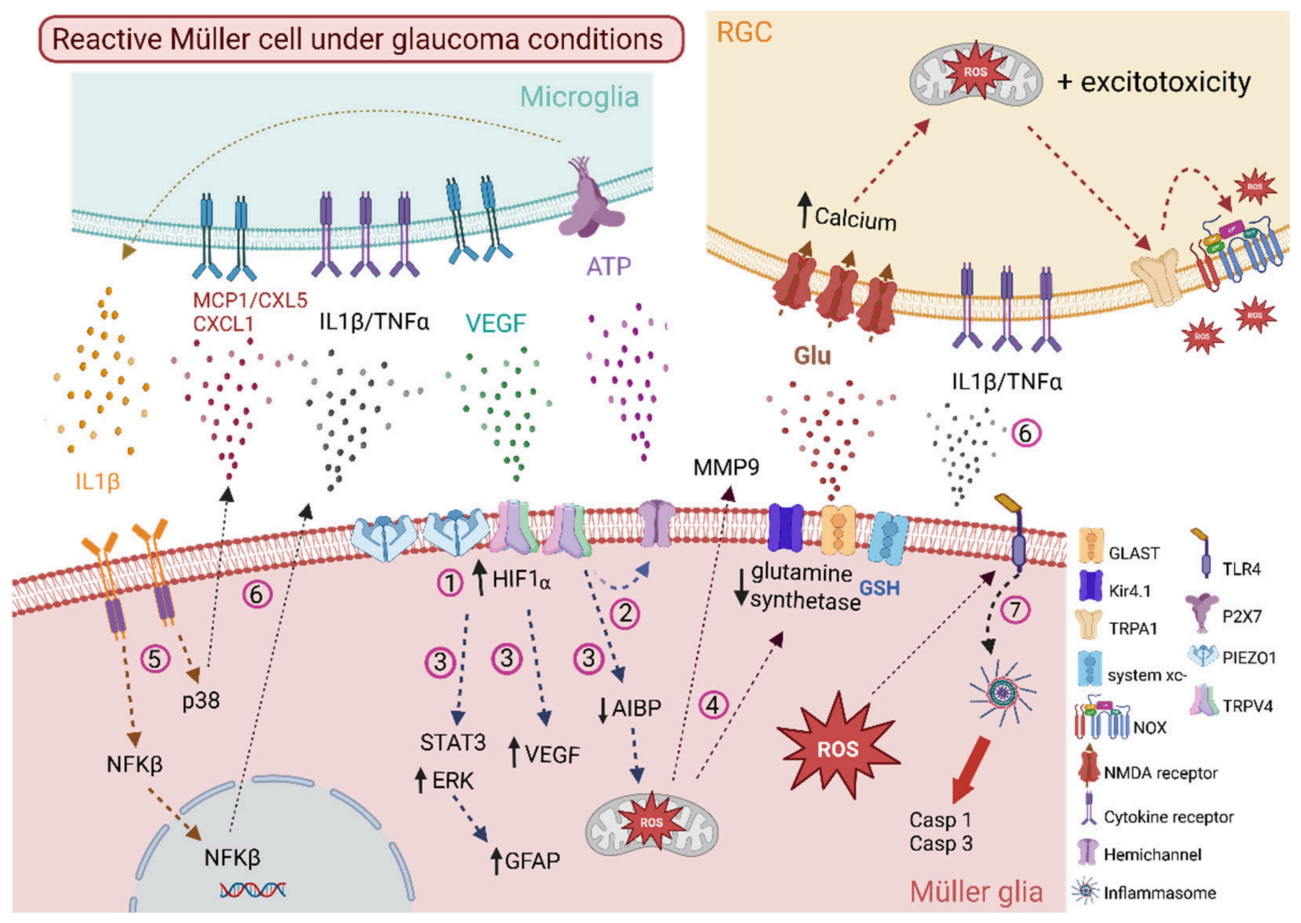
Signaling pathways and neurotoxic crosstalk of Müller cells in a glaucomatous context. Biomechanical stress induces the activation of mechanosensitive channels (Piezo1, TRPV4), promoting the upregulation of HIF-1α (1). This process leads to the inhibition of AIBP, inducing mitochondrial dysfunction and ROS accumulation (2), reinforced by the decrease in system x_c_^−^ and consequent reduction in glutathione (GSH), the main neuronal antioxidant. The activation of ERK and STAT3 by increased ROS levels leads to the upregulation of VEGF and GFAP, characterizing reactive gliosis (3). Concurrently, there is a downregulation of glutamine synthetase (GS), glutamate-aspartate transporter (GLAST) and Kir4.1 potassium channels, leading to extracellular glutamate accumulation, NMDA receptor overstimulation, and retinal ganglion cell excitotoxicity of retinal ganglion cells (RGCs) (4). In response to the microenvironment, activated microglia secrete IL-1β, stimulating Müller cell receptors and activating the p38 and NF-κB pathways (5). The translocation of NF-κB triggers the release of pro-inflammatory chemokines (MCP-1, CXCL5, CXCL1) and cytokines (IL-1β, TNF-α) (6). Increased oxidative stress activates TRPA1 channels and induces the formation of inflammasomes, with the subsequent activation of caspases 1 and 3 (7). ATP release through hemichannels stimulates microglia via P2X7 receptors, while excess glutamate hyperactivates NMDA receptors on RGCs. This promotes calcium influx and excitotoxicity, which, together with inflammation and oxidative stress, culminates in neurodegeneration. In the diagram, dashed arrows indicate downstream signaling pathways, molecular translocations, or cellular secretions, while solid arrows represent direct activation or upregulation. The colored dots represent secreted extracellular mediators (such as cytokines, chemokines, ATP, and glutamate). The greenish, yellow, and pinkish-red backgrounds designate the microglial, retinal ganglion cell (RGC), and Müller glial compartments, respectively. The blue structure within the Müller glia represents its nucleus. Created in BioRender. Calaza, K. D. (2026), https://BioRender.com/hwabt4z (accessed on 27 July 2026).

Alongside this early reactivity, the continuous mechanical stretch of Müller cell membranes stabilizes HIF-1α independently of oxygen levels (Figure 4) [122]. This mechanotransduction process upregulates HIF-1α expression at both the mRNA and protein levels in a response associated with the activation of transcription factors triggered by extracellular stress, such as NF-κB, driving its intracellular accumulation under normoxic conditions [122]. This mechanically induced HIF-1α stabilization shifts the cellular angiogenic profile by markedly upregulating the secretion of VEGF-A (Figure 4) while simultaneously downregulating the vessel-stabilizing angiopoietin-1 (ANG1) [122]. This reactive transformation is an early, initiating event in glaucoma pathogenesis rather than a late-stage secondary consequence. Histopathological analyses of human post-mortem glaucomatous retinas confirm that widespread Müller cell hypertrophy and GFAP overexpression occur autonomously and independently of the absolute extent of RGC loss [123]. Thus, mechanically activated Müller cells do not merely respond to neuronal death but actively orchestrate the neuroinflammatory microenvironment that accelerates glaucomatous damage (Figure 4).

### 4.3. Excitotoxicity and Oxidative Stress

Although the early response of reactive Müller glia is beneficial, chronic mechanical stress and severe cytoskeletal remodeling ultimately disrupt their functional polarity [106]. This structural collapse forces spatial mislocalization and downregulation of critical clearance mechanisms, precipitating a robust decrease in potassium buffering capacity [106]. The impaired potassium siphoning directly depolarizes the glial membrane, weakening the driving force for glutamate uptake by GLAST, which forces extracellular glutamate accumulation and induces ganglion cell excitotoxicity (Figure 4) [124,125].

Consistent with this, overexpression of Kir4.1 channels in a chronic ocular hypertension model inhibited Müller cell activation, the consequent microglial gliosis and inflammatory response, protecting ganglion cells [101]. A cytotoxic profile of Müller glia is also characterized by downregulation of the glutamate recycling enzyme GS (Figure 4), which also occurs in glaucoma models [126,127], accompanied by the reduction in GS activity [128]. This enzymatic failure further elevates extracellular glutamate [129], exacerbating excitotoxic ganglion cell death [124,129]. Accordingly, one of the animal models of glaucoma used in the literature is the intraocular injection of NMDA receptor agonists [130,131]. The increased activation of ionotropic glutamate receptors, especially NMDA receptors on RGCs, generates a massive intracellular calcium increase that leads to neuronal mitochondrial dysregulation [132], one of the main early sources of reactive oxygen/nitrogen species (ROS/RNS) (Figure 4) and a hallmark of glaucoma [133]. This redox imbalance arises when ROS overproduction overwhelms cellular antioxidant defenses. Notably, elevated IOP directly triggers this oxidative burden [134,135,136], leading to pathological protein modifications, such as the oxidation of GS [137], which disrupts important survival mechanisms.

In alignment with these findings, total antioxidant status is lower in open-angle glaucoma [138] and exfoliation glaucoma patients [139]. Moreover, this pro-oxidant state downregulates GLAST, crucial to extracellular glutamate concentration control, and xCT, the catalytic subunit of the x_c_^−^ transporter (Figure 4) [140], both important for the synthesis of glutathione [141]. Consequently, there is a marked reduction in glutathione in glaucoma models [142]. Therefore, the accumulation of free radicals establishes a vicious cycle that augments excitotoxicity and accelerates cell death. Furthermore, Müller gliosis induced by glaucoma models is accompanied by cell death, and treatments that prevent the reactivity of the Müller glia protect against neuronal loss [125,143,144]. These data reinforce the crucial role of redox dysregulation in Müller cells in driving the observed ganglion cell death in glaucoma.

### 4.4. Mitochondrial Collapse and TRP-Driven Oxidative Burden

The intersection between bioenergetic failure and innate immune activation represents a critical step in glaucomatous pathogenesis. This reactive transformation is not spatially uniform, as Müller cells located in the peripheral retina appear more susceptible to glaucomatous injury, exhibiting higher expression of TRPV4 and PIEZO1 than their central counterparts [116]. Rather than initiating mechanotransduction itself, sustained activation of these pathways contributes to intracellular calcium overload and progressive metabolic dysfunction. As a consequence, Müller cells undergo profound mitochondrial impairment characterized by downregulation of apolipoprotein A-I binding protein (AIBP), a key regulator of mitochondrial dynamics. AIBP deficiency promotes DRP1-dependent mitochondrial fragmentation, loss of mitochondrial membrane potential, and excessive mitochondrial ROS production (Figure 4) [145,146]. Metabolic exhaustion is further accompanied by reversal of glutamate transport, leading to extracellular glutamate accumulation [147], as well as increased MMP-9 secretion (Figure 4), which contributes to remodeling of the inner limiting membrane [147].

The resulting oxidative imbalance is further amplified by TRPA1, a highly sensitive redox sensor expressed in Müller and other retinal cells [148]. Activation of TRPA1 engages a NOX1-dependent pathway that further increases oxidative stress, promoting lipid peroxidation, including accumulation of 4-hydroxynonenal (4-HNE), and amplifying retinal inflammatory crosstalk (Figure 4). This feed-forward mechanism could disrupt glial-neuronal homeostasis, promote macrophage recruitment, and activate apoptotic signaling through caspase-3. Pharmacological inhibition or genetic deletion of TRPA1 probably interrupts this pathway by reducing NOX1 activation, limiting immune cell infiltration, and preserving retinal structure and RGC survival following oxidative injury [148].

TRPA1-dependent redox signaling may also be relevant to NTG, although direct evidence from NTG patients or NTG experimental models is currently lacking. Vascular dysregulation, unstable ocular perfusion, and recurrent ischemia/reperfusion injury have been proposed as pressure-independent contributors to NTG pathogenesis, providing a mechanistic reason to investigate TRP channels in this context [41,149]. Retinal ischemia/reperfusion models reproduce selected vascular and oxidative mechanisms associated with NTG without constituting disease-specific models. In these models, genetic deletion or pharmacological inhibition of TRPA1 reduces oxidative stress, inflammatory-cell recruitment, apoptotic signaling, and RGC loss [148]. Ischemia/reperfusion also increases TRPM7 expression in reactive Müller cells, but the causal contribution to retinal degeneration and RGC loss remains to be established [150]. Collectively, these findings raise TRPA1 as a potential mediator linking oxidative stress to neuroinflammation and support it as a therapeutic target in glaucoma [71].

### 4.5. Müller Glia as Central Drivers of Neuroinflammation

The massive accumulation of mtROS and damage-associated molecular patterns (DAMPs) serves as an endogenous danger signal that forces Müller glia to shift into a neurotoxic, senescence-associated inflammatory phenotype. By generating oxidized phospholipids (OxPLs) and altering membrane dynamics, the severe oxidative burden facilitates the rapid relocalization and membrane association of Toll-like receptor 4 (TLR4) on the Müller cell [145]. This newly membrane-associated TLR4 directly triggers the assembly of the NLRP3 inflammasome (leading to intracellular cleavage of caspase-1 and caspase-3) (Figure 4), while simultaneously activating the NF-κB and ERK signaling pathways [145,151]. This pro-inflammatory cascade initiates a highly coordinated, self-sustaining crosstalk with resident microglia. Acting as primary mechanosensors, reactive Müller cells release early danger signals (such as ATP) that recruit resident microglia to the injury site (Figure 4). Once activated, these early-responding microglia release massive amounts of IL-1β, which potently hyperactivates adjacent Müller glia via the NF-κB and p38 MAPK pathways (Figure 4), prompting a secondary secretory shift that releases chemokines like MCP-1 (CCL2), CXCL1, and CXCL5 [152]. These chemokines act as robust homing signals, binding to CXCR2 receptors on microglia and compelling their rapid migration directly into the ganglion cell layer. Thus, mechanosensitive Müller cells actively dictate the spatial recruitment of immune effectors, driving a toxic positive feedback loop that surrounds RGC axons with concentrated inflammatory signaling and accelerates irreversible visual dysfunction.

This pathogenic transformation of Müller glia is not confined to isolated cells; it propagates across the retinal tissue via Connexin-43 (Cx43) gap junctions and uncoupled hemichannels. While physiological Cx43 networks are vital for ion buffering and metabolic dissipation, elevated IOP triggers a massive pathological upregulation of Cx43 in Müller cells, notably occurring prior to the onset of RGC loss [153]. Under biomechanical and oxidative stress, these connexons frequently operate as uncoupled hemichannels, opening directly into the extracellular space. In an experimental glaucoma model, activated Müller cells utilized these Cx43 hemichannels to release copious amounts of ATP into the microenvironment [154]. The extracellular ATP functions as a danger-associated molecular pattern, binding to purinergic receptors (P2X7R/P2X4R) on resident microglia to forcefully drive their proliferation and migration toward the damaged retinal layers [154]. Through this mechanism, overexpressed Cx43 hemichannels act as conduits for the bystander effect, allowing mechanical injuries to leak inflammatory triggers that cascade into widespread neurodegeneration. Consequently, this interconnected web of macroglial reactivity, spanning from initial mechanosensation and oxidative collapse to Cx43-mediated inflammatory propagation, underscores that retinal glia actively coordinate glaucomatous damage.

Post-mortem studies of human glaucomatous eyes validate the presence of a pro-inflammatory microenvironment, highlighting significant elevations in retinal cytokines TNF-α, IL-1β, and IL-6, deeply intertwined with Müller cell reactivity [93]. Furthermore, fluid biomarkers reflect this neuroglial crosstalk: levels of Galectin-3 and APOE, key mediators of microglia–macroglia interactions, are elevated in the aqueous humor of glaucoma patients and correlate significantly with the historic maximum IOP [155], suggesting that mechanical strain on radial glia directly translates into an intraocular inflammatory signature. This inflammatory cascade appears to occur early in the disease process, as chemoattractants like IL-8 and vasoactive peptides like endothelin-1 (ET-1) are detectable in the aqueous humor even before severe clinical damage manifests [156].

Ultimately, the neuroinflammatory crosstalk driven by Müller glia, alongside the previously described astrocyte reactivity, establishes that macroglia are not mere secondary responders in glaucoma, but active drivers of retinal neurodegeneration. This pathogenic cascade, initially triggered by mechanosensors such as TRPV4 and amplified by profound oxidative stress, neuroinflammation, metabolic collapse and glial crosstalk, creates a self-sustaining neurotoxic microenvironment. Because this interconnected glial reactivity can perpetuate neuroinflammation and apoptotic signaling even after the initial mechanical strain is relieved, retinal damage frequently progresses despite intraocular pressure normalization. The key cellular interactions, signaling pathways, and functional outcomes that comprise this complex neuroglial network are summarized in Table 1. Therefore, directly interrupting this pathological macroglial network, particularly by targeting primary mechanosensitive and redox sensors, represents a fundamental paradigm shift for neuroprotection, laying the groundwork for advanced pharmacological interventions.

Summary of key cellular interactions, primary signal/receptor pairs, downstream signaling pathways, and functional outcomes among microglia, astrocytes, Müller glia, and retinal ganglion cells (RGCs).

## 5. Pharmacological Therapies

As mentioned before, current glaucoma treatments primarily aim to normalize IOP. Topical pharmacological therapy remains the first-line treatment and includes prostaglandin analogs, carbonic anhydrase inhibitors, and α2-adrenergic agonists. However, combined pharmacological therapies, as well as surgical interventions and laser procedures, can also be employed [159]. In some cases, the IOP reduction achieved by these treatments does not prevent glaucoma progression. Furthermore, many patients with glaucoma do not exhibit elevated IOP, suggesting that disease development and progression involve additional factors that remain to be fully understood. These findings reinforce the need to explore alternative therapeutic approaches that extend beyond IOP control. In this context, the regulation of retinal glial reactivity has emerged as a promising therapeutic target, together with the modulation of TRP channel activity.

TRP comprises a group of ion channels with similar molecular structures that are permeable to monovalent and divalent ions, with most members exhibiting high calcium permeability. The stimuli responsible for the activation of these receptors are diverse and may include thermal stimuli, plasma membrane stretching, endogenous/exogenous chemical molecules, pathogen-derived fragments, and inflammatory signals [71]. To date, seven TRP subfamilies have been described, including Ankyrin (TRPA), Canonical (TRPC), Melastatin (TRPM), Mucolipin (TRPML), NO-mechano-potential C (TRPN), Polycystin (TRPP), and Vanilloid (TRPV) [10.1007/978-981-10-7757-9_6] [160]. Although TRP channels were initially described in somatosensory neurons, it is now well established that they are expressed in different cell types throughout the CNS, including the retina, where they play both physiological and pathological roles [71]. Indeed, in glaucoma, there is evidence demonstrating the involvement of TRPV4 and TRPV1 receptors in retinal ganglion cell loss [161], although the knockout of each of these receptors did not protect retinal cells against cell death induced in the IOP-reperfusion model [148]. Intriguingly, only a few studies have investigated the relationship between TRP receptors and glial reactivity in animal models of glaucoma [15,161].

Animal models of glaucoma have demonstrated an important role for TRP channels in glial reactivity, as well as in the onset and progression of disease-associated symptoms. Among the wide variety of TRP channels, TRPV4 receptors have attracted particular attention. TRPV4 is a mechanosensitive non-selective cation channel that has been described in the retinas of different species and is expressed and functionally active in both neuronal cells and Müller glial cells [118,162]. Chronic elevation of intraocular pressure in mice and rats has been shown to induce Müller glial reactivity, which was associated with increased TRPV4 protein expression in these cells and enhanced retinal ganglion cell death [115,118]. The effects of chronic elevation of intraocular pressure were mimicked by intravitreal administration of GSK101, a TRPV4 agonist, and were prevented by prior treatment with HC-067, a TRPV4 antagonist, or R7050, a soluble TNF-α inhibitor [118]. Pro-inflammatory response-associated genes, such as IL-6, TNF-α, and STAT-3, were also found to be upregulated after 4 weeks of elevated intraocular pressure [115]. Thus, retinal ganglion cell loss in this model appears to depend on Müller glial reactivity and TNF-α release. A similar response has also been observed in in vitro models. Primary cultures and immortalized Müller glial cell lines became reactive following 24 h of activation of TRPV4 receptors with GSK101 [115,118,158,163]. Activation of TRPV4 receptors in glial cells led to increased TNF-α production and release, which may directly induce retinal ganglion cell death [118,158,164]. Cell death was inhibited by the addition of an anti-TNF-α antibody to the culture medium, indicating that TRPV4 activation-induced gliosis promotes the production and release of TNF-α [158]. The increased production and release of TNF-α are dependent on activation of the JAK/STAT-2 signaling pathway and phosphorylation of the NF-κB p65 subunit [118,158]. Activation of TRPV4 in retinal ganglion cell cultures also reduced cell viability and increased the expression of the TNF-α receptor TNFR1 [118,158]. This scenario supports the hypothesis that elevated intraocular pressure may lead to TRPV4 activation in both Müller glial cells and retinal ganglion cells. In Müller glia, TRPV4 activation induces glial reactivity and promotes the establishment of a pro-inflammatory profile that results in TNF-α release. In retinal ganglion cells, the expression of TNFR1 receptors is increased. In this context, enhanced TNF-α release combined with increased expression of its receptor in retinal ganglion cells appears to be a key factor driving the pro-inflammatory phenotype of Müller glia and the subsequent loss of retinal ganglion cells.

An important question that arises is how elevated intraocular pressure may lead to increased TRPV4 receptor expression in Müller glial cells. A possible clue to this question may come from the relationship between aquaporins and TRPV4 receptors. Aquaporins are among the most abundant water channels in the central nervous system (CNS) and play an important role in water homeostasis in Müller glia, where, together with TRPV4, they regulate membrane permeability and the regulatory volume decrease response induced by cell swelling [164,165]. However, recent studies have shown that aquaporin-4 and TRPV4 can physically interact, and that this relationship may be detrimental in both in vivo and in vitro models of diabetic retinopathy. Interestingly, inhibition of aquaporin-4 expression by shRNA prevents high-glucose-induced damage in a manner dependent on the reduction in TRPV4 expression. A similar effect has also been observed in streptozotocin-induced diabetic rats [166]. Thus, Müller glial cell death in diabetic retinopathy models depends on increased aquaporin-4 expression and the associated upregulation of TRPV4. On the other hand, activation of TRPV4 receptors and the subsequent activation of the PI3K/AKT signaling pathway in Müller glial cells by osteopontin, an integral component of the extracellular matrix, in osmotic swelling models reduce membrane permeability and the regulatory volume decrease response by decreasing aquaporin-4 expression [165]. These findings reveal a close interaction between aquaporin-4 and TRPV4 under different pathological conditions. To date, this relationship between TRPV4 and AQP4 has not been demonstrated in animal models of glaucoma, nor have the molecular mechanisms underlying this regulation been elucidated. It is conceivable that AQP4-induced cellular swelling leads to membrane stretch and subsequent activation of TRPV4 [164]. Calcium influx through this channel may further enhance AQP4 expression, thereby establishing a positive feedback loop that promotes Müller glial reactivity. Moreover, calcium influx mediated by TRPV4 has been shown to increase AQP4 surface expression through a calmodulin-dependent mechanism in primary human cortical astrocytes exposed to hypothermic conditions [167]. It is plausible that TRPV4-dependent glial reactivity in experimental models of glaucoma is mediated by an indirect regulation of AQP4 expression through calcium influx, calmodulin-dependent signaling, and activation of the PI3K/Akt signaling pathway. Therefore, investigating this potential relationship and its implications for glaucoma pathology may be of considerable interest.

Activated Müller glia in glaucoma models may also release pro-survival factors capable of regulating TRPV4 activity and expression. For example, chronic elevation of intraocular pressure in animal models has been shown to increase the production and release of lipoxin B4 by Müller glial cells, thereby acting as a neuroprotective agent. Lipoxin B4 is an endogenous eicosanoid produced through the coordinated action of 5-lipoxygenase (5-LOX) and 15-lipoxygenase (15-LOX). Notably, lipoxin B4, as well as 5-LOX and 15-LOX, are expressed in mouse Müller glial cells [115]. Administration of lipoxin B4 was able to prevent the increase in TRPV4 expression, glial reactivity, and the inflammatory response induced in vivo and in vitro by elevated intraocular pressure or TRPV4 activation [115]. Another factor that may regulate TRPV4 activity is the amount of cholesterol present in the membrane. TRPV4 receptor activity induced by GSK101, temperature, and cellular swelling was reduced in Müller glial cells following membrane cholesterol depletion by MβCD [168]. Therefore, reducing TRPV4 receptor activation appears to be a promising therapeutic strategy.

Other members of the TRP family have also been identified as inducers of Müller glial reactivity. A brief 30 min ischemic event in the rat retina followed by 72 h of reperfusion led to an increase in TRPM7 immunoreactivity in Müller glial cells, but not in astrocytes. Upregulation of TRPM7 channels following ischemia–reperfusion was accompanied by a reduction in the amplitude of oscillatory potentials in ERG recordings, indicating alterations in the inner retina, as well as mild decreases in b-wave and nSTR amplitudes [150]. TRPV1 activation also plays an important role in retinal ganglion cell loss in glaucoma models [161]; however, only a few studies have sought to understand its relationship with Müller glial cell activation. Cultured mouse Müller glial cells proliferate and release ATP in response to increased hydrostatic pressure (12–48 h) in a TRPV1- and PLCγ-1-dependent manner [16]. Retinal ganglion cells exposed to conditioned medium from Müller glial cells subjected to increased hydrostatic pressure undergo cell death in a manner dependent on the ATP receptor P2X7. Altogether, the findings suggest that TRPV1-induced ATP release from Müller glia leads to P2X7 activation in retinal ganglion cells, which subsequently undergo autophagy-dependent apoptotic cell death [16]. On the other hand, rat Müller glial cells apparently do not express TRPV1 [169]. Nevertheless, Müller glial activation observed 7 days after injury in the optic nerve axotomy model appears to depend indirectly on TRPV1 activation, as well as on the production of NO and peroxynitrite [169]. However, retinal damage persisted in TRPV1- or TRPV4-knockout animals following 60 min of intraocular pressure (IOP)-induced ischemia (I) and 2–7 days of reperfusion (R), indicating that under these experimental conditions these receptors are not essential for the induction of cell death [148]. In contrast, retinas from TRPA1-knockout animals are resistant to I/R injury [148]. Pharmacological blockade of TRPA1 with HC-030031 was also able to inhibit cell death induced by 50 min of oxygen–glucose deprivation (OGD) in the embryonic chicken retina [170]. Activation of TRPA1 receptors appears to play an important role in retinal ischemic events. However, in these studies, the authors did not evaluate glial reactivity in this context or its dependence on TRPA1 receptor activation. OGD increased TRPA1 protein expression in the retinas of the animals [170]. This increase may be occurring in Müller glial cells and could be responsible for triggering a pro-inflammatory response that promotes cell death.

Although substantial evidence links TRP receptors to Müller glial cell function, there are currently no studies in the literature associating these receptors with astrocyte activation in glaucoma models. Nevertheless, single-cell RT-PCR analyses have demonstrated that several TRP isoforms (TRPC1-2, TRPC6, TRPV2, TRPV4, TRPM2, TRPM4, TRPM6-7, and TRPP1-2), as well as Piezo receptors (Piezo1-2), are expressed in astrocytes of the optic nerve head, with most astrocytes expressing TRPC1 and TRPP1 [70]. The lack of information regarding the role of TRP receptors in astrocytes represents an excellent opportunity to further investigate these receptors in glaucoma models.

Taken together, the available evidence reveals a broad involvement of TRP receptors in Müller glial activation and cell loss across different pathological retinal models. To date, however, no studies have evaluated a possible association between TRPM7 expression and glaucoma models, nor the potential involvement of TRPV1 in this context. It is possible that Müller glial activation depends on the coordinated action of TRPV1, TRPV4, and TRPM7. Modulating Müller glial activation therefore represents an attractive strategy for slowing glaucoma progression, with TRP receptors emerging as promising therapeutic targets. Nevertheless, the expression profile of TRP channels in Müller glial cells from rodents and humans should be carefully considered when evaluating the translational potential of therapeutic strategies based on TRP channel inhibition. To date, no comprehensive studies have characterized the expression of TRP channels in human Müller glial cells. The available evidence suggests the presence of TRPV1 and TRPV4 [161], supporting the rationale for the development of future therapies targeting these channels. This hypothesis is further strengthened by the substantial body of preclinical evidence linking TRPV1 and TRPV4 signaling to retinal ganglion cell loss in experimental models of glaucoma.

## 6. Gene Therapy Approaches Targeting Macroglial Dysfunction in Glaucoma

As mentioned before, although IOP-lowering therapies remain the cornerstone of glaucoma management, retinal ganglion cell degeneration often progresses despite adequate IOP control, highlighting the contribution of IOP-independent mechanisms such as oxidative stress, neuroinflammation, and metabolic dysfunction. In this context, gene therapy has emerged as a promising strategy to provide long-term modulation of disease-associated pathways following a single administration. Unlike most current gene therapy strategies, which primarily target retinal ganglion cells or aqueous humor dynamics [171,172], macroglia-directed approaches seek to modify the retinal microenvironment by enhancing the homeostatic functions of Müller cells and astrocytes while limiting their chronic neurotoxic responses [2,171,173,174]. Compared with retinal ganglion cells, macroglia represent particularly attractive targets for gene therapy because they remain viable throughout most stages of glaucoma and establish extensive interactions with neurons, retinal vasculature, and immune cells. Müller cells span the entire retinal thickness, whereas astrocytes form an interconnected network surrounding retinal ganglion cell axons and the optic nerve head. Consequently, modulation of a relatively small population of glial cells may influence multiple pathological processes simultaneously, including metabolic support, glutamate recycling, oxidative stress, inflammatory signaling, and neurotrophic factor secretion. Rather than simply protecting injured neurons, macroglia-directed therapies have the potential to reprogram the retinal microenvironment toward a more neuroprotective state [85,175,176].

Importantly, the therapeutic goal should not be the complete suppression of reactive gliosis. As discussed throughout this review, macroglial activation is highly dynamic, with early responses often supporting tissue repair, antioxidant defense, glutamate clearance, and metabolic homeostasis, whereas prolonged activation promotes chronic inflammation, complement activation, and neuronal dysfunction. Therefore, future gene-based therapies should seek to preserve the beneficial aspects of macroglial activation while selectively attenuating pathways associated with persistent neuroinflammation [85,86,176].

### 6.1. Vector Tropism and Cell-Specific Gene Expression

Adeno-associated viral (AAV) vectors remain the leading platform for ocular gene delivery because of their favorable safety profile, relatively low immunogenicity, and long-term transgene expression [174,177]. However, successful macroglial targeting depends not only on the viral capsid but also on promoter selection, route of administration, retinal architecture, and species.

Among the available vectors, ShH10 and its modified variants have demonstrated preferential transduction of Müller glia after intravitreal administration in rodents, making them attractive platforms for glia-targeted therapies [178,179]. Nevertheless, these findings should be interpreted cautiously, as retinal anatomy, inner limiting membrane composition, and receptor distribution differ substantially between rodents, non-human primates, and humans, potentially altering vector tropism [174].

Promoter selection provides an additional level of specificity. The RLBP1 promoter has received considerable attention because it preferentially drives transgene expression in Müller glia and retinal pigment epithelial cells and has shown activity in rodent models, non-human primates, and human-derived retinal cells. Moreover, its successful use in clinical studies for inherited retinal diseases supports its translational potential [180,181]. However, RLBP1 is not exclusively Müller cell-specific, and its behavior in the glaucomatous human retina remains incompletely established.

Alternatively, the GFAP promoter may preferentially target reactive astrocytes and Müller cells, allowing disease-dependent expression, although it also lacks strict cellular specificity [85]. Together, these observations suggest that combining optimized viral vectors with cell-selective or disease-responsive promoters may represent the most effective strategy for achieving precise modulation of macroglial function in glaucoma.

### 6.2. Therapeutic Targets

Macroglia-directed gene therapy may either reinforce physiological glial functions or suppress maladaptive inflammatory responses. Candidate therapeutic genes include neurotrophic factors (such as BDNF, CNTF, and GDNF), antioxidant enzymes (including SOD2), and regulators of inflammatory pathways such as NF-κB, the NLRP3 inflammasome, complement signaling, and purinergic receptors [86,171,173,174]. Rather than completely inhibiting these pathways, future strategies will likely require a balanced modulation that preserves the neuroprotective functions of macroglia while preventing the chronic inflammatory phenotype associated with glaucoma progression. The choice of therapeutic target may also depend on disease stage, with early interventions favoring metabolic and antioxidant support and later stages requiring greater suppression of persistent inflammatory signaling [85,176]. Table 2 summarizes the principal macroglia-directed therapeutic targets discussed in this review, highlighting their proposed mechanisms of action, potential benefits, and current translational status.

### 6.3. Translational Challenges and Clinical Perspectives

Despite encouraging preclinical results, several barriers still limit the clinical translation of macroglia-directed gene therapy. Intravitreal administration, although less invasive than subretinal delivery, may induce neutralizing antibodies, complement activation, and local inflammatory responses that reduce transduction efficiency and complicate repeated dosing [174,183]. These challenges are expected to be even greater in larger eyes, where retinal penetration differs substantially from that observed in rodents.

Current efforts to overcome these limitations include engineered AAV capsids, inducible promoters, transient immunosuppression, and non-viral delivery systems [174]. Nevertheless, glaucoma presents additional complexity because of its multifactorial nature and prolonged course. Consequently, macroglia-directed gene therapy is unlikely to replace conventional IOP-lowering treatments but may become an important adjunctive neuroprotective strategy for patients who continue to progress despite adequate pressure control.

Another major issue in the disease course is determining in vivo the timeframe when macroglia shifts from an adaptive, neuroprotective state to a maladaptive, neurotoxic phenotype. Clinically, glaucoma is typically diagnosed through structural changes in the fundus and RNFL. However, by the time these macroscopic alterations are detectable, the molecular shift to a neurotoxic glial phenotype may have already occurred [2,123]. Because glaucomatous damage is asynchronous, with different retinal and optic nerve regions exhibiting distinct stages of injury simultaneously, strictly chronological timelines are insufficient. Instead, establishing the therapeutic window relies on molecular profiling. Based on the mechanisms discussed, the adaptive phase is characterized by the preservation of homeostatic proteins, such as Kir4.1 and GS [101,127]. The precise moment of neurotoxic switch could be identified by the localized loss of functional molecular markers, coupled with the emergence of neurotoxic drivers.

In astrocytes, these include the upregulation of SASP components [79], the CXCL10/CXC3/C3 axis [80], and TRPC-mediated fibrotic remodeling in the lamina cribrosa [71]. In Müller glia, this switch is marked by AIBP downregulation [145,146], uncoupled Cx43 hemichannels [153], and TRPA1-driven oxidative stress [71]. Thus, this phenotypic switch dictates the therapeutic strategy. If interventions occur during an early adaptive phase, therapies can focus on metabolic and antioxidant support. However, once the switch is established, the provision of trophic support may be ineffective. In this case, interventions in this later window must silence these dominant neuroinflammatory, fibrotic, and senescence cascades to potentially prevent irreversible neuronal loss.

Future clinical translation will depend on the development of vectors capable of selectively targeting human macroglia, the identification of reliable biomarkers to define the optimal therapeutic window, and the demonstration of long-term efficacy and safety in chronic glaucoma models [173,174].

## 7. Conclusions

Astrocytes and Müller cells are essential for maintaining retinal homeostasis by providing structural, metabolic, and neurotrophic support. In glaucoma, however, persistent biomechanical, metabolic, and inflammatory stress progressively disrupts these homeostatic functions, driving glial dysfunction and contributing to retinal neurodegeneration. Because macroglial activation is initially protective but becomes detrimental when sustained, future therapeutic strategies should focus on modulating specific glial phenotypes rather than indiscriminately suppressing gliosis. Identifying the optimal therapeutic window for such interventions will be critical to maximize neuroprotection while preserving the physiological functions of macroglia.

Mechanosensitive channels, particularly members of the TRP family and PIEZO1, have emerged as important regulators of macroglial responses during glaucoma. Rather than acting solely as mechanotransducers, these channels integrate mechanical, metabolic, and oxidative signals that shape the transition from protective gliosis to chronic neuroinflammation. Although considerable progress has been made, important questions remain regarding the cell type-specific functions of these channels and the stage of disease at which their modulation would provide the greatest therapeutic benefit.

Among these channels, TRPA1 deserves particular attention because it links oxidative stress to persistent neuroinflammatory signaling. Since TRPA1 can be activated independently of mechanical stimuli by reactive oxygen species, it may also contribute to retinal degeneration in forms of glaucoma with minimal or no elevation in intraocular pressure, including NTG. Further investigation of this pathway may expand therapeutic opportunities beyond conventional IOP-lowering strategies.

In addition to the pathways discussed above, neurosteroids such as 17β-estradiol and DHEA-S have demonstrated neuroprotective effects in experimental retinal ischemia, possibly through σ1 receptor-mediated metabolic support. Likewise, pharmacological activation of σ1 receptors has been shown to protect retinal cells against oxidative stress, suggesting that modulation of endogenous neuroprotective mechanisms may represent an additional avenue for future investigation [184,185]. Although sex is not considered a risk factor for glaucoma, some evidence suggests that estrogen can protect retinal cells from insult [31,32], potentially by modulation of glial cells [33,34]. Thus, there is a possibility that menopause could act as a risk factor for glaucoma. Therefore, this is a very interesting question to be investigated.

Finally, important translational challenges remain before macroglia-targeted therapies can be successfully implemented in clinical practice. These include achieving precise cell type-specific targeting, ensuring controlled transgene expression, and minimizing immune responses to viral vectors. Despite these limitations, increasing evidence indicates that selective modulation of macroglial function represents a promising therapeutic strategy to slow glaucomatous neurodegeneration and complement current pressure-lowering treatments.

## Figures and Tables

**Table 1:** Intercellular signaling pathways and outcomes in retinal glia–RGC interactions.

Cellular Interaction	Signal/Receptor	Downstream Pathway	Functional Outcome
Microglia → astrocytes	IL-1α, TNF-α and C1q	NF-κB activation	Cytotoxic astrocyte polarization and loss of neuroprotective functions [87,90]
Injured RGCs/microglia → astrocytes	CXCL10/CXCR3	C3 upregulation	Maintenance of neurotoxic astrocyte reactivity [80]
Astrocytes → RGCs	C3a/C3aR	Complement signaling	Acceleration of RGC apoptosis [80]
Microglia → Müller glia	IL-1β	NF-κB and p38 MAPK	Pro-inflammatory activation and chemokine release [152]
Müller glia → microglia	CXCL1 and CXCL5/CXCR2	Chemotactic signaling	Migration toward the ganglion cell layer [152]
Müller glia → microglia	ATP/P2X7R and P2X4R	Purinergic signaling	Microglial activation, proliferation, and migration [154]
Müller glia → RGCs	ATP/P2X7	Ca^2+^ influx and P2X7R-mediated cell death	RGC death [157]
Müller glia → RGCs	TNF-α/TNFR1	Pro-inflammatory signaling	Reduced viability and neuronal death [118,158]
Müller glia → RGCs	BDNF/TrkB	PI3K/AKT	Neuronal survival and protection [105]
Astrocytes → RGCs	CNTF	Neurotrophic signaling	Protection against oxidative damage [59,67]
Müller glia → RGCs	Glutamate/NMDA receptors	Ca^2+^ overload and mitochondrial dysfunction	Excitotoxicity and RGC death [124,129,130,131,132]
Astrocytes ↔ Müller glia	Cx43 gap junctions	Intercellular signal propagation	Intercellular coupling and propagation of glial signals across the retina [5,92]

**Table 2:** Overview of candidate gene therapy strategies targeting macroglial dysfunction in glaucoma. Proposed molecular targets, mechanisms of action, expected therapeutic effects, preferred stage of intervention, and current translational status are summarized.

Vector/Promoter or Strategy	Predominant Target	Evidence/Model	Potential Application to Glaucoma	Main Translational Limitation	References
AAV6-derived ShH10	Müller glia	Intravitreal delivery in mice	Delivery of neuroprotective or anti-inflammatory cargos from Müller cells	Tropism established mainly in rodents; uncertain efficiency and selectivity in primates	[171,178,179]
ShH10Y	Müller glia-enriched	Mouse retina and human Müller cells in vitro	Enhanced Müller glial transduction	Limited in vivo validation in human-relevant glaucoma models	[174,179]
AAV9	Müller glia and other retinal cells, depending on route and promoter	Experimental retinal models	Broad retinal delivery combined with a cell-selective promoter	Incomplete cellular specificity and possible off-target expression	[171,182]
RLBP1 promoter	Müller glia and retinal pigment epithelium	Mouse, non-human primate and human-cell studies; clinical use in RLBP1 retinal dystrophy	Sustained expression of antioxidant or neurotrophic factors	Not exclusively Müller-specific; glaucoma-directed efficacy unproven	[180,181]
GFAP promoter	Reactive Müller glia and astrocytes	Activated mouse retinal glia	Disease-responsive expression in areas of gliosis	Expression depends on activation state and is not cell-exclusive	[85,174]
Hypoxia-responsive glial promoter	Müller glia in metabolically stressed retinal areas	Experimental hypoxic retinal models	Spatially and temporally restricted expression in ischemic or metabolically compromised glaucoma	Not validated in chronic glaucoma	[171,174]
Glial expression of secreted trophic factors, e.g., GDNF, BDNF or CNTF	Müller glia/astrocytes as producer cells	Preclinical retinal neurodegeneration models	Paracrine protection of RGCs and axons	Dose control, receptor desensitization and potential off-target effects	[171,173,174]
RNA interference or CRISPR repression of NF-κB, NLRP3, C3 or P2X7R	Reactive macroglia	Primarily conceptual or preclinical pathway evidence	Reduction in chronic neuroinflammatory amplification	Risk of suppressing beneficial immune and reparative functions	[86,174,176]
Enhancement of SOD2, glutathione pathways, Kir4.1 or glutamate handling	Müller glia/astrocytes	Mechanistic and preclinical evidence	Restoration of redox, ionic and neurotransmitter homeostasis	Long-term overexpression may disturb normal retinal physiology	[10,86]
Non-viral nanoparticles or RNA delivery	Dependent on formulation and targeting ligands	Early preclinical retinal studies	Redosable and potentially less immunogenic modulation	Lower durability, limited penetration and inefficient endosomal escape	[171,174]

## Data Availability

No new data were created or analyzed in this study. Data sharing is not applicable to this article.

## Abbreviations

The following abbreviations are used in this manuscript:

4-HNE	4-hydroxynonenal
AIBP	Apolipoprotein A-I binding protein
ANG1	Angiopoietin-1
AQP4	Aquaporin-4
BDNF	Brain-derived neurotrophic factor
BRB	Blood–retinal barrier
C3	Complement component 3
CNS	Central nervous system
CNTF	Ciliary neurotrophic factor
Cx43	Connexin-43
DAMP	Damage-associated molecular pattern
DDR	DNA damage response
ECM	Extracellular matrix
ERG	Electroretinogram
ERK	Extracellular signal-regulated kinase
ET-1	Endothelin-1
GCL	Ganglion cell layer
GFAP	Glial fibrillary acidic protein
GLAST	Glutamate/aspartate transporter
GS	Glutamine synthetase
GSDMD	Gasdermin D
GSH	Glutathione
H_2_O_2_	Hydrogen peroxide
HIF-1α	Hypoxia-inducible factor-1 alpha
IL	Interleukin
ILM	Inner limiting membrane
IOP	Intraocular pressure
LOX	Lipoxygenase
MHC	Major histocompatibility complex
MMP	Matrix metalloproteinase
mtROS	Mitochondrial reactive oxygen species
NF-κB	Nuclear factor kappa B
NLRP3	Inflammasome
NO	Nitric oxide
NOX	NADPH oxidase
NTG	Normal-tension glaucoma
O_2_	Superoxide radical
OGD	Oxygen–glucose deprivation
OLM	Outer limiting membrane
ONH	Optic nerve head
OXPHOS	Oxidative phosphorylation
PEDF	Pigment epithelium-derived factor
RGC	Retinal ganglion cell
RNFL	Retinal nerve fiber layer
RNS	Reactive nitrogen species
ROS	Reactive oxygen species
SASP	Senescence-associated secretory phenotype
SOD	Superoxide dismutase
TCA	Tricarboxylic acid
TGF-β2	Transforming growth factor-β2
TGM2	Transglutaminase 2
TLR4	Toll-like receptor 4
TNF-α	Tumor necrosis factor alpha
TRP	Transient receptor potential
TRPA1	Transient receptor potential ankyrin 1
VEGF	Vascular endothelial growth factor
xCT	Cystine/glutamate antiporter subunit
YAP	Yes-associated protein
